# Supplementary material for: Taxonomy annotation and guide tree errors in 16S rRNA databases
Source: PeerJ. 2018 Jun 12;6:e5030. doi: 10.7717/peerj.5030 (PMC6003391; doi:10.7717/peerj.5030)

Minimal subtree of LTP tree release 128 for Rhodobacter.

This is the smallest subtree containing all 11 LTP sequences for genus Rhodobacter. The subtree contains 390 sequences from 122 different genera. Each leaf is labeled with its sequence accession number and genus name. Rhodobacter sequences are shown by red text.

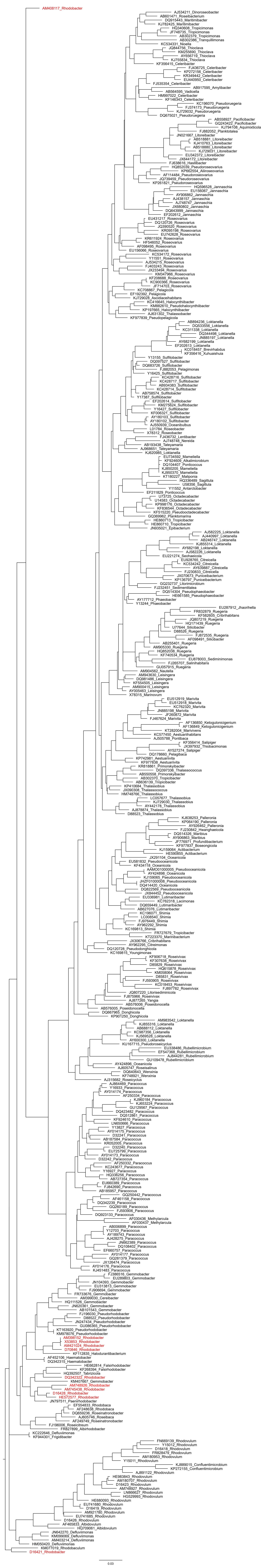

Supplement: Supplemental Information 1 — The LTP subtree under its Rhodobacter LCA node, which contains 390 sequences from 122 genera. [file peerj-06-5030-s001.pdf]
